# US States’ Childhood Obesity Surveillance Practices and Recommendations for Improving Them, 2014–2015

**DOI:** 10.5888/pcd13.160060

**Published:** 2016-07-28

**Authors:** Kelly J. Blondin, Catherine M. Giles, Angie L. Cradock, Steven L. Gortmaker, Michael W. Long

**Affiliations:** Author Affiliations: Catherine M. Giles, Angie L. Cradock, Steven L. Gortmaker, Harvard Prevention Research Center, Harvard Chan School of Public Health, Boston, Massachusetts; Michael W. Long, Department of Prevention and Community Health, Milken Institute School of Public Health, The George Washington University, Washington, DC.

## Abstract

**Introduction:**

Routine collection, analysis, and reporting of data on child height, weight, and body mass index (BMI), particularly at the state and local levels, are needed to monitor the childhood obesity epidemic, plan intervention strategies, and evaluate the impact of interventions. Child BMI surveillance systems operated by the US government do not provide state or local data on children across a range of ages. The objective of this study was to describe the extent to which state governments conduct child BMI surveillance.

**Methods:**

From August through December 2014, we conducted a structured telephone survey with state government administrators to learn about state surveillance of child BMI. We also searched websites of state health and education agencies for information about state surveillance.

**Results:**

State agency administrators in 48 states and Washington, DC, completed telephone interviews (96% response rate). Based on our interviews and Internet research, we determined that 14 states collect child BMI data in a manner consistent with standard definitions of public health surveillance.

**Conclusion:**

The absence of child BMI surveillance systems in most states limits the ability of public health practitioners and policymakers to develop and evaluate responses to the childhood obesity epidemic. Greater investment in surveillance is needed to identify the most effective and cost-effective childhood obesity interventions.

## Introduction

The dramatic rise in childhood obesity in the United States during recent decades is well-documented (1–3). Height and weight surveillance data were critical in detecting the epidemic and motivating efforts to address it. However, few data are available to assess trends in small geographic areas. No surveillance system used to track childhood obesity nationally collects state-level measured data on height and weight. Biennially the National Health and Nutrition Examination Survey (NHANES) collects measured anthropometric data on a nationally representative, but not state representative, sample of children aged 0 to 19 years (4). For more than 3 decades, the Pediatric Nutrition Surveillance System provided state-level measured data on roughly 8 million children aged 0 to 5 years in low-income households that participated in federal maternal and child health programs, but that survey was discontinued in 2012 (5). The National Survey of Children’s Health collects parent-reported data on 95,000 children aged 0 to 17 years (6), and the Youth Risk Behavior Surveillance System collects self-reported data from high school students in most states (7); both of these surveys collect state-representative data, but they rely on reported height and weight, which are less accurate than measured data (8,9).

Previous research on states’ measurement of child body mass index (BMI) has focused on identifying state surveillance legislation, describing best practices, and reviewing controversial school-based BMI screening programs (10–16). In 2010, one study reported that 19 states had enacted BMI surveillance laws and regulations, but implementation of these policies lagged (10). Another study found that in 2012, 25 states had legislation requiring school-based BMI screening, but the study did not examine the extent of policy implementation (13).

The aim of this study was to assess US states’ child BMI surveillance practices and provide guidance for best practices. To our knowledge, this is the first systematic evaluation of state practices related to childhood obesity surveillance.

## Methods

We surveyed US states to learn about their practices related to childhood obesity surveillance. Consistent with established definitions of public health surveillance (17), we defined a BMI surveillance system as a program that meets 4 criteria: 1) height and weight are measured and reported by a trained professional; 2) data are state representative for the age groups included in the survey or census sample; 3) data are collected at least every 2 years with plans to continue; and 4) data are aggregated, analyzed, and reported at the state level.

From August through December 2014, we called the state health agency in all 50 states and Washington, DC, and asked to speak with the person most knowledgeable about child BMI surveillance practices in the state. We invited the identified key informant to participate in a telephone interview via an initial telephone call and up to 3 additional telephone calls or emails. If unable to reach the target individual, we attempted to identify an alternate informant and repeated the outreach and follow-up protocol.

We interviewed the individual identified as the most appropriate contact, using a structured interview guide developed to solicit information about state BMI measurement practices. We asked respondents whether they knew of any efforts in the state to routinely collect and analyze child height and weight data, and we asked a series of questions to determine whether the state’s practices met any or all of the criteria we used to define BMI surveillance. The interview included questions about state laws pertaining to physicals children undergo before starting school, state attempts to use electronic health records (EHRs) as a source of surveillance data, and any existing or pending legislation mandating collection of data on child BMI. The interview guide also included questions about funding sources and other resources required to implement a BMI surveillance program. We verified the accuracy and completeness of survey responses through a search of publicly available information on the websites of each state’s health and education agencies.

This study was approved by the Office of Human Research Administration at the Harvard Chan School of Public Health.

## Results

Our outreach resulted in telephone survey responses from key informants in 48 of the 50 states and Washington, DC (96% response rate). For the 2 states from which we did not receive a response, we identified a surveillance system in 1 state (New Mexico) and a school-based BMI measurement program without a data reporting component in the other (New Jersey) from information on the states’ health agency websites.

We found that 14 states (27%) conduct BMI surveillance consistent with the definition applied in this study (Figure) and that those states collect child height and weight data through several common approaches. Five states collect BMI data through a school-based survey, in which data on child health indicators are assessed at school for surveillance purposes only. Three states measure BMI as part of a school-based physical fitness test to assess selected measures that produce a composite score for physical fitness. Five states conduct BMI surveillance by using data collected through a school-based health screen, after which parents or guardians are informed of their child’s measurement. A single state collects data on a randomly selected representative sample outside the school setting (Table 1). Our survey found that state government administrators in 5 states were aware of their state conducting pilot evaluations of EHR-based child BMI surveillance. 

**Figure:**
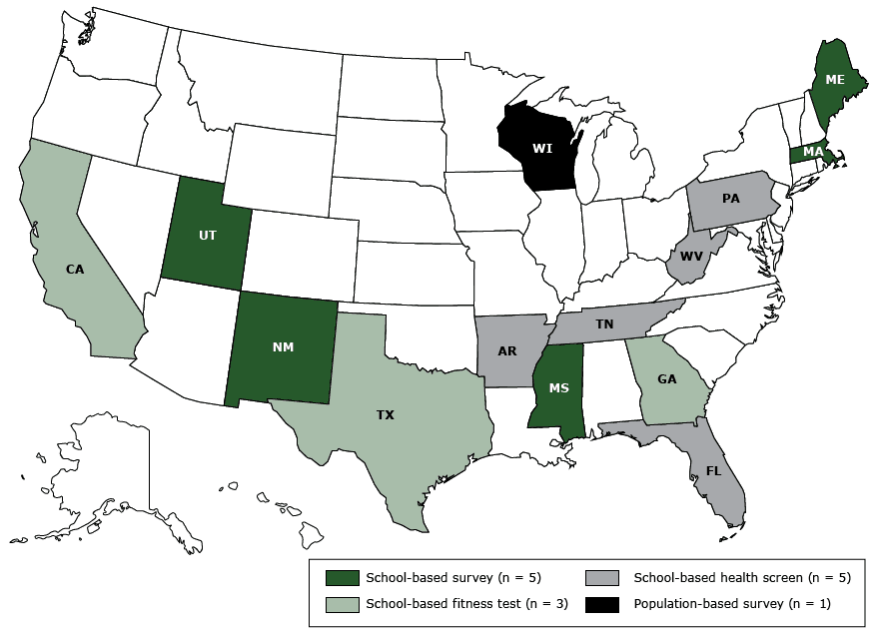
State childhood obesity surveillance systems in the United States, 2014–2015. **Table:** 

Surveillance Method	State(s)
School-based survey	Maine, Massachusetts, Mississippi, New Mexico, Utah
School-based fitness test	California, Georgia, Texas
School-based health screen	Arkansas, Florida, Pennsylvania, Tennessee, West Virginia
Population-based survey	Wisconsin

**Table 1:** Surveillance Practices of the 14 States That Have Surveillance Systems for Collecting Data on Child Body Mass Index (BMI), United States, 2014–2015^a^

State	Method^b^	Sample type^c^	Policy Mandate^d^	Grades Surveyed	No. of Years Data Collected	Survey Interval	Longitudinal Data^e^	Local Data^f^	Sample Size^g^
**Arkansas**	School health screen	Census	Yes	Even K–10	11	Annual	Yes	Yes	178,631
**California**	School fitness test	Census	No	5, 7, 9	17	Annual	Yes	Yes	1,335,931
**Florida**	School health screen	Census	Yes	1, 3, 6	7	Annual	No	Yes	484,694
**Georgia**	School fitness test	Census	Yes	1–12	3	Annual	Yes	Yes	1,139,998
**Maine**	School survey	Sample	Yes	K, 3, 5	3	Biennial	No	Yes	11,484
**Massachusetts**	School survey	Census	Yes	1, 4, 7, 10	3	Annual	No	Yes	205,975
**Mississippi**	School survey	Sample	No	K–12	5	Biennial	No	No	480,321
**New Mexico**	School survey	Sample	No	K, 5	3	Annual	Yes	No	3,949
**Pennsylvania**	School health screen	Census	Yes	K–12	7	Annual	Yes	Yes	1,803,689
**Tennessee**	School health screen	Census	No	Even K–8, one high school year	7	Annual	No	Yes	276,877
**Texas**	School fitness test	Census	Yes	3–8, one high school year	8	Annual	Yes	Yes	2,903,200
**Utah**	School survey	Sample	No	1, 3, 5	5	Biennial	No	No	4,477
**West Virginia**	School health screen	Census	Yes	2	10	Annual	No	Yes	8,591
**Wisconsin**	Population survey	Sample	No	Not reported	1	Annual	No	Not reported	Not reported

a These states have child BMI measurement programs that satisfy the criteria for a surveillance system: 1) height and weight are measured and reported by a trained professional; 2) data are state-representative for the age groups included in the survey or census sample; 3) data are collected at least every 2 years with plans to continue; and 4) data are aggregated, analyzed, and reported at the state level.

b A school-based survey entails measuring child health indicators at school for surveillance purposes only; a school health screen involves measuring health indicators to inform parents/guardians of their child’s health status; a school fitness test is designed to assess selected measures to produce a composite score of overall physical fitness; and a population survey collects data on a randomly selected representative sample drawn from the population of interest.

c A census sample includes all children in a population of interest; a sample is a randomly selected subgroup of children selected or retrospectively weighted to represent a population of interest.

d Policy mandate for fitness testing or BMI screening but not BMI surveillance.

e Longitudinal data refers to BMI data collected on the same children over time; each child’s data are linked by a unique identifier.

f Local-level data were reported by 9 of the 14 states; 5 reported school-level data, 6 reported school district–level data, and 5 reported county-level or public health district–level data.

g Sample or census size based on most recently published data.

Nine states attempt to take a census of all children in public schools in specific grades, and the other 5 states collect data from a representative sample of children. Coverage across grade levels among states conducting school-based BMI surveillance ranged widely, from a single grade in 2 states to 13 grades in 2 states (mean, 6 grades). Eleven of the states collect data annually and 3 states biennially. Eleven states collect and report locally representative data in addition to state data.

Although only 14 states maintain a surveillance system meeting our study’s criteria, most states (n = 35) collect child height and weight data. Twenty-one states reported routinely collecting child BMI data but to an extent not fulfilling our standard for surveillance (Table 2). In 7 states, the child BMI data collected are not state representative because, for example, districts collect and report data voluntarily. Six states do not aggregate, analyze, and report the child BMI data they collect, and 8 states collect child BMI data less often than every 2 years (on average, every 5 years). An additional 8 states conducted BMI surveillance at least once, many in the past 2 to 3 years, and some had plans to repeat the surveys.

**Table 2:** US States^a^ (n = 21) That Collect Data on Child Body Mass and Meet at Least One Criterion for a Surveillance System but Do Not Have a Surveillance System, 2014–2015

State	Method	Surveillance Criteria Met^b^			
		Height and Weight Measured by a Trained Professional	Data Are Collected at Least Every 2 Years	Data Are State Representative	Data Are Aggregated, Analyzed, and Reported
**Alabama**	School-based oral health screen/survey	X		X	X
**Alaska**	School-based oral health screen/survey	X	X		X
**Arizona**	School-based oral health screen/survey	X		X	X
**Colorado**	School-based oral health screen/survey	X		X	X
**Hawaii**	School entry physical	X	X	X	
**Idaho**	School-based survey	X		X	X
**Illinois**	School-based oral health screen/survey	X		X	X
**Kentucky**	School entry physical	X	X		X
**Louisiana**	School-based fitness test	X	X		X
**Minnesota**	School entry physical	X	X	X	
**Nebraska**	School health screen	X	X	X	
**New Hampshire**	School-based oral health screen/survey	X		X	X
**New Jersey**	School-based screen	X	X	X	
**New York**	School-based survey	X	X		X
**North Carolina**	School entry physical	X	X	X	
**North Dakota**	School-based oral health screen/survey	X		X	X
**Ohio**	School-based oral health screen/survey	X		X	X
**Oklahoma**	School-based fitness test	X	X		X
**South Dakota**	School-based survey	X	X		X
**Virginia**	School entry physical	X	X	X	
**Washington, DC**	School entry physical	X	X		X

a Among 50 states and Washington, DC (n = 51).

b Criteria for a surveillance system: 1) height and weight are measured and reported by a trained professional; 2) data are state-representative for the age groups included in the survey or census sample; 3) data are collected at least every 2 years with plans to continue; and 4) data are aggregated, analyzed, and reported at the state level.

Of the 14 states, 10 have legislation requiring child BMI surveillance. Pending legislation was not reported by any interviewees. Administrators in 6 states provided estimates of costs related to conducting BMI surveillance, and another 6 cited insufficient funding as an impediment to initiating, maintaining, or fully implementing surveillance efforts.

## Discussion

Our evaluation of US states’ childhood obesity surveillance practices found that only 14 operate BMI surveillance programs. States with surveillance programs used the data to detect disparities in the prevalence of overweight and obesity based on socioeconomic status and race/ethnicity. For example, Li and colleagues analyzed more than 1 million BMI records collected on children in grades 1, 4, 7, and 10 between 2009 and 2014 in Massachusetts and found a decline in obesity prevalence overall, but no improvement among children in low-income districts (18). Similarly, Drewnowski and colleagues’ analysis of BMI data collected during a census of children in grades 5, 7 and 9 revealed a significant association between California Assembly District poverty status and childhood overweight prevalence (19). Madsen and colleagues also used data from California’s statewide school-based BMI measurement program to explore the prevalence of overweight and obesity by race/ethnicity and sex. Their analysis of data collected between 2001 and 2008 on more than 8 million students revealed growing disparities whereby black, Hispanic, and American Indian children were more likely than non-Hispanic white and Asian children to have a high BMI, and black and American Indian girls were the only groups not to show improvement in overweight or obesity (20).

Surveillance data also show risk and protective factors for childhood obesity. For example, Alviola and colleagues analyzed BMI data for Arkansas children in even grades kindergarten through 10 during the 2008–2009 school year and reported an association between a school’s proximity to fast food and the prevalence of obesity among its students (21). Likewise, Sage and colleagues used child BMI data collected in Texas public schools to investigate how neighborhood characteristics such as access to fast food, fresh produce, and recreation space relate to neighborhood-level obesity prevalence (22).

Child BMI surveillance data can also be used to assess the impact of childhood obesity interventions on obesity prevalence. Surveillance data collected in Massachusetts, Texas, and California are used to evaluate the effectiveness and cost-effectiveness of multisector, multicomponent demonstration projects in those states (23). Given the value of child BMI data for understanding and mitigating the childhood obesity epidemic, the current shortage of state-level child BMI surveillance data undermines progress.

The practices of the 14 states conducting child BMI surveillance offer guidance on surveillance methods that can meet various budgets and public health planning needs. Ideally, state-level child BMI surveillance systems entail annual data collection on a large sample or census of children of a range of ages. Furthermore, longitudinal data (repeated measures collected on the same children over time) greatly enhance the ability to detect small but meaningful shifts in population-level BMI. Surveillance systems with these qualities provide the best data for identifying disparities in obesity trends, understanding determinants of local variation in obesity prevalence, and evaluating public health interventions locally. Three states (Georgia, Pennsylvania, and Texas) have exemplary surveillance programs; mandated by state laws, they collect BMI data annually on a census sample of children at 10 or more grade levels and can link data on individuals across years at the local level.

However, limited public health resources may hinder optimal state BMI surveillance. By including fewer grade levels, conducting surveys of representative samples, or collecting data every other year, states can conduct meaningful surveillance at a lower cost. Combining BMI measurement with an existing child health surveillance initiative such as the school-based oral health screening program is a cost-efficient surveillance approach (11).

Although school-based measurement is the most common approach for child BMI surveillance, it is not without limitations. Research has identified potential negative effects of collecting height and weight data in schools, including stigma, bullying, body image dissatisfaction, and disordered eating (16). Controversy surrounds the parental notification component central to BMI screening programs (24) and school-based BMI measurement generally (25). Other potential limitations include the cost of data collection, burden on school staff, potentially unreliable data quality, and exclusion of preschool-aged children and children in home or private schools. Some states are exploring alternative settings for collecting BMI surveillance data. Wisconsin is piloting in-home BMI data collection as part of a population-based random sample survey modeled on NHANES. Although this approach reduces the burden on schools and students, unless the sample is large and representative at the school district and/or city level, it will have limited utility for evaluating local trends and the impact of local policy changes and may be expensive.

Interest in using electronic health records (EHR) as a BMI surveillance system is increasing. Since 2009, BMI measurement is a requirement of the Healthcare Effectiveness Data and Information Set, a tool used by more than 90% of US healthcare plans to measure provider performance (26,27). Moreover, the HITECH Act of 2009 authorized incentive payments to increase physicians’ adoption of EHR systems (28). However, EHR uptake by pediatric practices lags uptake by other practice types. As of 2012, only 14% of pediatric practices had adopted fully functional EHR systems and only 8% had adopted systems fully supportive of pediatric practice needs, including well-child visit tracking and anthropometric analyses (27,28). However, even universal adoption of EHR systems would not provide complete coverage of child and adolescent populations because of infrequent pediatric visits during later childhood, lack of medical care access among some sociodemographic groups, and frequent change of providers. Nevertheless, given expanded healthcare coverage under the Affordable Care Act and the frequency of pediatric visits, state public health officials will probably continue to explore the potential of EHR-based child BMI surveillance.

This study has several limitations. Although it is a review of state-level child BMI surveillance practices, it was limited by a lack of information on surveillance program costs and integrity. The survey questions about program costs and resource use did not yield adequate information to produce meaningful cost estimates. Only 6 states operating surveillance programs were able to provide qualitative or quantitative information about program costs during the interview. We learned that funding shortages often undermine surveillance programs and reduce the resources available to operate them; an in-depth analysis of the costs of various surveillance options should be conducted so that policymakers can weigh these costs against the potential benefits. In addition, we were unable to evaluate the quality of height and weight measurement practices in states conducting surveillance, a concern raised by the public health community (29).

Although we found that only 14 states have a child BMI surveillance system according to the definition applied in this study, many states conduct some degree of BMI surveillance. Many states recently conducted their first child BMI surveillance, suggesting that gaps in state data may be filled as states respond to the childhood obesity epidemic. Given recent evidence that the steep upward trend in childhood obesity rates in the United States may be leveling, at least among certain sociodemographic groups (19,30), public and political support for action may dwindle even though obesity rates are high and continue to increase among some groups. Particularly concerning is that national statistics, which suggest that childhood obesity rates may be leveling, may divert attention from disparities in child BMI according to socioeconomic status or race/ethnicity. State and local surveillance is better than national surveillance at capturing data on prevalence in local communities to identify those disproportionately affected.

Increasing investment in state and local child BMI surveillance is crucial to maintaining attention on the burden of childhood obesity in local communities. Locally representative child BMI data are also needed to evaluate the impact of policy and program efforts to reverse the epidemic. The public health community must make surveillance a priority to guide the investment of scarce intervention resources. Without a substantial increase in surveillance, policymakers have insufficient data to identify the most effective and cost-effective responses to the childhood obesity epidemic.
